# Pathogenetic Impact of Bacterial–Fungal Interactions

**DOI:** 10.3390/microorganisms7100459

**Published:** 2019-10-16

**Authors:** Filomena Nogueira, Shirin Sharghi, Karl Kuchler, Thomas Lion

**Affiliations:** 1 CCRI—St. Anna Children’s Cancer Research Institute, Vienna 1090, Austria; shirin.sharghi@ccri.at; 2 Labdia—Labordiagnostik GmbH, Vienna 1090, Austria; 3 Center of Medical Biochemistry, Max Perutz Labs, Campus Vienna Biocenter, Medical University of Vienna, Vienna 1030, Austria; karl.kuchler@meduniwien.ac.at; 4 Department of Pediatrics, Medical University of Vienna, Vienna 1090, Austria

**Keywords:** bacterial–fungal interactions, microbiome, molecules, immune response, *in vivo* models

## Abstract

Polymicrobial infections are of paramount importance because of the potential severity of clinical manifestations, often associated with increased resistance to antimicrobial treatment. The intricate interplay with the host and the immune system, and the impact on microbiome imbalance, are of importance in this context. The equilibrium of microbiota in the human host is critical for preventing potential dysbiosis and the ensuing development of disease. Bacteria and fungi can communicate via signaling molecules, and produce metabolites and toxins capable of modulating the immune response or altering the efficacy of treatment. Most of the bacterial–fungal interactions described to date focus on the human fungal pathogen *Candida albicans* and different bacteria. In this review, we discuss more than twenty different bacterial–fungal interactions involving several clinically important human pathogens. The interactions, which can be synergistic or antagonistic, both *in vitro* and *in vivo*, are addressed with a focus on the quorum-sensing molecules produced, the response of the immune system, and the impact on clinical outcome.

## 1. Introduction

Identification and subsequent exploitation of the mechanisms that mediate communication between bacteria and fungi, as well as the interplay with the host and the immune system, can provide the basis for the discovery of novel biomarkers facilitating improved diagnostics and more effective treatment approaches. *In vivo* models are required for clinically relevant understanding of bacterial–fungal interactions (BFI), as currently available *in vitro* assays may not reflect the implications in the affected host. In some instances, the findings of BFI were even completely discrepant between different experimental settings by revealing antagonistic interactions *in vitro* and synergistic effects in *in vivo* models, presenting with increased virulence and pathogenicity in the host [1,2]. Current diagnostic approaches are not designed to routinely assess polymicrobial infections, and may therefore miss the presence of bacterial–fungal co-infections. In instances in which the co-infection results in one-way or mutual inhibitory interactions, treatment with antibiotics or antifungals may therefore promote or unleash growth of the microbiota not covered by the antimicrobial therapy, with possible implications for morbidity and mortality. Studies have shown that mixed cultures of *Candida* and bacteria occur in about 23% of the reported episodes of candidemia, with a predominance of *Staphylococci* in these associations [3]. However, no significant differences in the clinical outcome of patients with mixed bloodstream infections were observed in comparison to monomicrobial candidemia. Prior use of antibiotics was associated with decreased prevalence of mixed *Candida*–bacterial bloodstream infections [3]. The clinical implications of bacterial–fungal interactions are reviewed in [4,5,6,7], and there is evidence that some BFI can promote disease [8]. Associations between *Candida* and *Enterobacter* have been detected in all types of clinical specimens investigated and, in most cases, the frequency of these associations was statistically significant. Co-isolation of *Enterococcus faecalis* and fungi was reported in 22% of patients treated in intensive care units, and other bacteria frequently co-isolated with fungi included the genera *Klebsiella* and *Serratia* [7]. Co-infections by *Candida* and the bacteria *Enterobacter*, *Klebsiella*, or *Serratia* have led to increased rates of hospitalization [3,7]. Another study showed that a significant proportion of candidemias occurred in parallel with bacteremia, although no significant differences in long-term survival were observed between single and co-infections [3]. Kett and colleagues have shown that 38% of patients testing positive for *Candida* infection had a co-infection with bacteria [9]. Similarly, postmortem blood analysis showed mixed infections by *Candida* and bacteria in 39% of the cases investigated [10].

Communication between bacteria and fungi is mediated via quorum sensing molecules and proteins [11,12,13]. Identification of these signalling molecules and assessing their interplay with the immune system, e.g., by determining specific cytokine profiles, renders them an attractive target for more specific diagnostics and treatment. Recent studies have identified a set of signaling molecules secreted by human intestinal microbiota that accumulate at detectable concentrations, and have suggested that these molecules could be used as markers of disease (reviewed in [14]). Microbial and, specifically, bacterial–fungal interactions, can also result in the production of molecules potentially affecting host homeostasis. Moreover, recent insights derived from metagenomic and metabolomic analyses indicate that the integration of multifaceted data can provide the basis for improved therapies [15,16]. Krüger and colleagues have described fungal–bacterial interactions with a focus on the mucosal niches and consequences for the human host [17]. In the present review, we focus on describing the spectrum of BFI identified to date, with potential relevance for the human host. The focus is placed on the mechanisms of intermicrobial communication, including the respective metabolites involved and the interplay with the host immune system, addressing the cytokine and chemokine profile of BFI.

## 2. Bacterial–Fungal Interactions in the Context of the Microbiome

Microbial interactions are an integral part of the highly complex human microbiome. Mapping of the human microbiome has shown a wide diversity of bacteria and fungi occupying specific niches [18,19,20,21]. Whereas most studies have focused primarily on the bacteriome, the realms of fungi and viruses, the mycobiome and virome, have been studied less extensively. The human intestinal mycobiome has a considerably lower diversity compared to the bacteriome. The dominant genera in a healthy individual include *Saccharomyces, Malassezia*, and *Candida* [19]. Microbes may have beneficial, neutral, or harmful effects while interacting with the host and the immune system. The microbiota play a key role in host homeostasis, including the regulation of the immune system and production of essential vitamins, amino acids, metabolites, and byproducts necessary for the normal function of many processes [22,23,24,25,26,27]. Intestinal microbiota also protect against colonization by pathogenic microorganisms.

However, once the equilibrium of the microbiota is disturbed, e.g., by a variety of conditions or alterations, including diet, treatment with antibiotics or other drugs, age, stress, chronic inflammation, or various underlying diseases, shifts in the levels and composition will occur, with consequences for the onset and progression of disease [18,26,27,28]. Variations in the intestinal microflora have been helpful in establishing differences between the states of health and disease [16,18,20,29,30,31,32,33,34,35,36,37,38,39].

Experimental approaches addressing the microbiome have been based on the use of completely germ-free (gnotobiotic) mice to assess the impact of an altered microbiome from diseased mice transplanted into these animals [40,41,42]. It was shown that the disease-associated microbiome is commonly adopted by the gnotobiotic mice, facilitating studies on the role of specific microbes and their composition on metabolic and immune processes [43,44,45,46,47]. Such studies in mouse models have contributed to improved understanding of the influence of an imbalanced microbiome on the health status, and have set the stage for studies addressing the complex microbial interactions occurring in the human host. Bacteria and fungi often live in organized structures, termed biofilms, rather than in planktonic state. The formation of either intra-domain (bacterial–bacterial or fungal–fungal) or inter-domain (bacterial–fungal) interactions within biofilms have been implicated in a variety of diseases, such as cystic fibrosis (e.g., interactions between *Inquilinus limosus*, *Dolosigranulum pigrum*, *Burkholderia cepacia* and *Pseudomonas aeruginosa*), endocarditis, prostatitis, and cancer [48,49]. Bacterial biofilms have been suggested to play a role in the progression of colorectal cancer (CRC) [50], and polymicrobial, i.e., bacterial–fungal co-infections (e.g., interactions between *Candida albicans*, *Aspergillus fumigatus*, and *P. aeruginosa*) were shown to display more deleterious effects in the context of cystic fibrosis compared to single pathogen infections [51,52,53]. Similarly, the fungal genera *Candida* and *Rhodotorula* have been linked to atopic diseases, including asthma in infants [54,55]. By contrast, certain bacterial–fungal interactions, such as those of various *Candida* species and *Lactobacilli,* which are part of the normal vulvovaginal microflora, were demonstrated to be beneficial for the host by preventing candidiasis at this site [56,57]. Moreover, *Candida* is also thought to prevent life-threatening urinary tract infections by *Escherichia coli* [58,59,60].

### 2.1. Impact of Microbiome Dysbiosis on the Bacterial–Fungal Equilibrium

Dysbiosis is characterized by changes in the amount, composition, distribution, function, and metabolic activity of physiological microbiota. It is associated with loss of biodiversity and overgrowth of pathogenic species [39]. A variety of very diverse diseases have been associated with dysbiosis, including inflammatory bowel disease, obesity, allergy, diabetes, autism, and colorectal cancer, where dysbiosis can either be a causal factor or a secondary effect of the disease [39]. Bacterial dysbiosis, displaying the prevalence of completely different types of bacteria in comparison to healthy lungs, has been described in chronic lung diseases such as asthma, chronic obstructive pulmonary disease (COPD), or cystic fibrosis [18]. In Crohn´s disease (CD), dysbiosis with increased levels of Proteobacteria, Fusobacteria, and the fungal species *C. albicans* and *Candida glabrata*, has been described during disease progression [36,61]. Moreover, commensal fungi such as *Saccharomyces cerevisiae* may also display harmful effects during dysbiosis by inducing damage of the intestinal barrier [62]. Antibiotic or antifungal treatment may have implications for the balance and the interactions between bacteria and fungi. For example, depletion of commensal intestinal fungi may unleash the growth of bacteria with pathogenic potential, leading to an exacerbation of colitis [63]. Colonization with *C. albicans* in mice treated with antibiotics was shown to increase allergic airway disease [64], but antifungal treatment was also reported to mediate a similar clinical effect [65]. Hence, in various states of dysbiosis, loss of the bacterial–fungal equilibrium and corresponding interactions can play a pathogenetic role in different diseases.

### 2.2. Microbial Metabolites—the Good and the Bad

Intestinal microbes can communicate with the host via microbial metabolites, which may mediate beneficial or harmful effects [66]. The host benefits from microbiota owing to their production of certain amino acids and vitamins [25,67], and commensal microbes also produce a range of small molecules inhibiting the growth of pathogenic microorganisms. For example, specific intestinal bacteria such as Firmicutes produce short-chain fatty acids (SCFA), including butyrate, acetate, and propionate, through the fermentation of fibers and other polysaccharide compounds, which have an important role in immune development, control of inflammation, and defense against infection [68]. Butyrate is also used as an energy source for intestinal epithelial cells, thereby conferring protection against pathogens [69]. Butyric acid also inhibits the yeast-to-hyphal transition of *C. albicans*, a key virulence attribute of this opportunistic pathogen [70], and several studies have associated butyrate with protective effects against infectious diseases. However, the net effect of a microbial metabolite can depend on the specific environmental conditions. Butyrate was shown to display protective effects against colorectal cancer (CRC), but a decrease in the butyrate-producing bacteria Firmicutes was described in inflammatory bowel disease (IBD), thus potentially compromising the beneficial effect of this SCFA [71,72]. However, other studies performed in a murine model described butyrate as promoting carcinogenesis by enhancing epithelial cell proliferation [73]. The controversial effects of butyrate are well documented [74]. Other examples of harmful metabolites include alanine and lactate, which are present in the lungs of cystic fibrosis patients, and promote the growth of *P. aeruginosa* [75]. Furthermore, phenolic compounds, amines, ammonia, acetaldehyde, nitrosamines, and sulphides are microbial metabolites generated by protein fermentation, which have been shown to be toxic for the host (e.g., by impairing metabolic functions or by mediating DNA damage), and also promoting of cancer development [35,38,76,77]. Production of such toxins in the intestinal tract is mediated by quorum sensing communication among different microbial species [28].

## 3. Communication between Bacteria and Fungi Mediated by Proteins and Small Molecules

**Figure 1:**
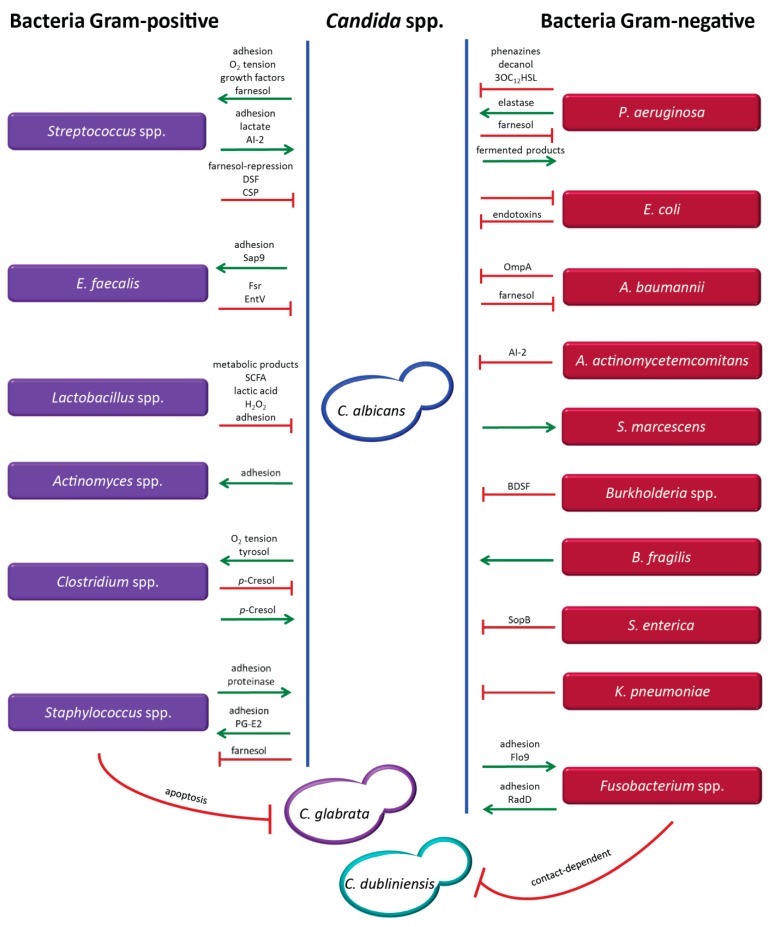
Molecules and factors mediating the interaction between different *Candida* species and a variety of bacteria. *Candida* species include *Candida (C.) albicans, Candida (C.) glabrata* and *Candida (C.) dubliniensis*. Gram-positive bacteria are represented in lilac (*Enterococcus (E.) faecalis*) and Gram-negative bacteria in red (*Pseudomonas (P.) aeruginosa, Escherichia (E.) coli, Acinetobacter (A.) baumannii, Aggregatibacter (A.) actinomycetemcomitans, Serratia (S.) marcescens, Bacteroides (B.) fragilis, Salmonella (S.) enterica, Klebsiella (K.) pneumoniae*). Green arrows indicate supportive interactions and red lines represent inhibitory effects. If not indicated above the green arrows and red lines, the molecules mediating the interaction are currently unknown.

Microbial interactions are mediated by several mechanisms that also serve as virulence factors, including quorum sensing, biofilm formation, production of secondary metabolites, and cellular signal transduction [78,79,80,81,82,83]. Such interactions can be quite complex, and occur particularly when different microorganisms share the same niches in the host, resulting in differential effects that can be antagonistic, synergistic, or neutral. Hence, bacteria and fungi can mutually support their growth or exert competitive effects, potentially leading to suppression of one microorganism and dominant growth of the other. Suppressive and inhibitory interactions mediated by different molecules or factors can occur simultaneously, depending on specific stimuli and changes in the microenvironment (Figure 1). Several studies have demonstrated that polymicrobial infections can be more severe and result in considerably higher mortality than infections with single pathogens, as reported, for example, for co-infections with *A. fumigatus* and *P. aeruginosa* or with *C. albicans* and *P. aeruginosa* in certain clinical settings [1,2,51,52,53,84,85,86]. New therapeutic strategies targeting quorum sensing molecules and bacterial virulence factors are being tested with the aim to deliver more efficient antimicrobial treatment and to prevent development of drug resistance [87,88,89,90,91]. Such approaches were tested specifically against infections with *P. aeruginosa* and were able to disrupt the cell communication and to reduce the virulence [92,93,94,95]. Other studies have attempted to use antibiotics and antifungals in combination with quorum sensing molecules to treat bacterial–fungal infections [96]. For example, the quorum sensing molecule farnesol, which inhibits filamentation of *C. albicans*, [97] has been tested in combination with antifungal drugs, and resulted in decreased minimal inhibitory concentration (MIC) values [98].

The bacterial–fungal interactions described to date mainly involve the interplay of *C. albicans* with different bacterial species. Exploitation of the growing knowledge of the microbiome offers new insights into the diversity of bacterial and fungal species colonizing the human body, many of which share the same niches. This information, along with pertinent clinical studies, unravels microbial interactions of potential clinical relevance. Here, we describe the mechanisms involved in various bacterial–fungal interactions in the human host, with a focus on quorum sensing molecules and virulence factors.

### 3.1. Bacterial–Fungal Interactions

#### 3.1.1. *Candida* Species and Different Bacteria

*Candida albicans* and *Pseudomonas aeruginosa* (Figure 1): This bacterial–fungal interaction is one of the most widely studied microbial interplays. Well documented examples of sites revealing an interplay between these pathogens include intravenous catheters, lungs of cystic fibrosis patients, the respiratory tract of ventilated patients, and burn wounds [11]. The interaction between *C. albicans* and *P. aeruginosa*, which is mediated by the production of quorum sensing molecules and virulence factors, is rather complex, as synergistic and antagonistic effects can occur simultaneously [2,86,99,100,101,102,103,104]. *P. aeruginosa* produces phenazines, including pyocyanin as a toxic end product, decanol, and 3-oxo-C12-homoserine lactone (3OC_12_HSL), which inhibit *C. albicans* biofilm formation and hyphal development via generation of highly toxic reactive oxygen species (ROS) [2,86,99,100,101,102,103,105,106]. Interestingly, the concentrations of homoserine lactone (HSL) were considerably higher in biofilms than in the presence of these microbes in planktonic state [107]. Additional molecules produced by *P. aeruginosa* include hemolytic phospholipase C and other virulence factors, such as GacA, LasR, RhlR, and RpoN [108]. The capacity of *P. aeruginosa* to adhere to the hyphal form of *C. albicans* is 30 times higher than binding to the yeast form, resulting in condition-dependent killing of hyphae [84,108]. In addition to secreting inhibitory molecules, *P. aeruginosa* can also increase the virulence of *C. albicans* by producing the proteolytic enzyme elastase (LasB), thus underscoring the differential effects mediated by *P. aeruginosa* in this interaction [100]. Conversely, farnesol, a quorum-sensing molecule produced by *C. albicans,* can downregulate the quorum sensing system of *P. aeruginosa* by affecting the production of pyocyanin and reducing bacterial motility [109]. To the advantage of *P. aeruginosa*, generation of fermented products by *C. albicans* enhances the phenazine secretion by *P. aeruginosa,* thus promoting colonization of the lungs by the bacterium [110]. More details on this interaction have been described previously [111]. Several studies reported that co-colonization by *C. albicans* and *P. aeruginosa* occurs at a statistically significant frequency and results in a decrease of pulmonary function, leading to inferior clinical outcome. Although the mortality rates in some *in vivo* models including mouse and zebrafish were elevated, possibly as a result of exacerbated inflammatory response, the differential outcomes in various animal models are still a matter of controversy [1,2,51,52,86]. The effects of this interaction may also depend on the specific colonizing strains and the immune status of the host. Thus, more studies are needed to assess the potential importance of this BFI in the clinical setting.

*Candida albicans* and *Streptococcus* spp. (Figure 1): The strong adherence and synergistic interaction between different *Streptococcus* species and *C. albicans* promoting stable formation of biofilms is well documented, and favors survival and enhanced colonization by these microbes, particularly in the oral cavity and the gastrointestinal tract [112,113]. The adhesins Als1, Als3 and Als5 (agglutinin-like sequence) produced by *Candida* are important for aggregation and adhesion to bacterial cells [114,115,116], while the binding of *Streptococci* is mediated by the cell surface polypeptide CshA and the antigen I/II salivary adhesins SspA and SspB [115]. *C. albicans* induces the growth of various *Streptococcus* species, including *S. oralis, S. gordonii, S. sanguinis, S. mutans*, by stimulating the formation of adhesion sites, reducing the oxygen tension, providing growth factors (e.g., polysaccharides) generated by its metabolic activity, and by inducing biofilm formation of *Streptococcus* spp. via farnesol production [117,118,119]. Importantly, mixed biofilms of *C. albicans* and *Streptococcus* display increased resistance to antifungal and antibiotic treatments [120]. Conversely, *Streptococcus* spp. can promote growth of *C. albicans* by producing lactate, which can be exploited as a carbon source by the fungus [117,121]. Moreover, *Streptococcus* species promote adhesion of *C. albicans* by expressing polysaccharide receptors and polypeptide adhesins. Additionally, *Streptococci* can stimulate hyphal development via secretion of the quorum sensing molecule AI-2 (autoinducing peptide) and by repression of the *C. albicans*-derived quorum sensing molecule farnesol, which functions by suppressing hyphal formation at high cell density [122]. Interestingly, a recent study has shown that isolates of *C. albicans* from patients with recurrent vulvovaginal candidiasis show attenuated hyphal formation in the presence of *S. agalactiae* [123]. Finally, *Streptococcus* species including, specifically, *S. oralis* can also promote dissemination of *C. albicans* to distal organs by currently unknown mechanisms [113]. However, depending on the environmental conditions, *Streptococci* can also inhibit hyphal formation through a diffusible signal factor (DSF) and the competence-stimulating peptide (CSP). The factor DSF, a *trans*-2-decenoic acid, is an intermediate product of unsaturated fatty acid synthesis. This molecule is related to farnesoic acid and farnesol, which are quorum-sensing molecules of *C. albicans* inhibiting filamentation. However, the mechanism of DSF secretion remains unclear. The molecule CSP is only produced during the early exponential phase of growth, and has been shown to increase biofilm formation, acid tolerance, and production of bacteriocin, a peptide toxin [124,125,126]. Despite the potentially differential effects mediated by the interaction of these microorganisms, the net result is most commonly mutual promotion with strong biofilm formation, suggesting that higher dosages of antimicrobial drugs may be required to control or eradicate the infection.

*Candida albicans, Candida glabrata*, and *Staphylococcus* spp. (Figure 1): These microorganisms are responsible for a considerable proportion of hospital infections, and are often co-isolated from urinary tract catheters and in a variety of conditions, including buccal mucositis, cystic fibrosis, keratitis, pneumonia, and wound infections [127,128,129]. The pathogen *Staphylococcus aureus* is reportedly the third most common bacterial species co-isolated with *C. albicans* [130]. Adhesion of *S. aureus* to *C. albicans* creates a more extensive biofilm, particularly when this bacterium binds to the hyphal form, which displays 30-fold higher adhesion rates compared to the yeast form [84]. Biofilm formation in catheters in *in vivo* models of *S. aureus* infections is enhanced by the presence of *C. albicans* through the attachment of Als3 to *S. aureus* adhesins [84,131]. Prostaglandin (PG) E2 produced by *C. albicans* is involved in stimulating growth and biofilm formation of *S. aureus* in co-culture, and fungal cell wall polysaccharides secreted into the biofilm matrix increase the tolerance of *S. aureus* to antimicrobial treatment [127,132]. Moreover, *C. albicans* was shown to promote systemic dissemination of *S. aureus* to the kidneys in a murine model of oral co-colonization [131]. Conversely, *S. aureus* supports adhesion of *C. albicans* to the buccal mucosa via the production of proteinase [133]. However, in the presence of farnesol, either externally added into culture or secreted by *C. albicans,* the viability and capacity of biofilm formation of *S. aureus* were reduced, owing to the ability of farnesol to disrupt the cell membrane integrity of the bacterial pathogen [134]. This resulted in increased susceptibility to antibiotic treatment and impaired growth [135,136]. Another *Staphylococcus* species, *S. epidermidis*, adheres to both yeast and hyphal forms of *C. albicans,* and the extracellular matrix produced by *S. epidermidis* protects the fungus [100]. This association also results in increased resistance to antimicrobial drugs including, for example, fluconazole and vancomycin [100]. In contrast to the largely synergistic interactions outlined above, an antagonistic relationship of *S. aureus* towards *C. glabrata* conveyed by an apoptosis-mediated mechanism has recently been described. However, the molecular mediators of this effect have not been identified to date [137].

*Candida albicans* and *Enterococcus faecalis* (Figure 1): These microorganisms can be commonly isolated from a variety of clinical samples [138,139], and the bacterium secretes a compound that inhibits hyphal formation of *C. albicans* via the Fsr quorum sensing system. Two proteases expressed by Fsr, GelE (gelatinase, a metalloprotease II) and SerE (serine protease), play an important role in this process [140]. This inhibitory effect was also observed in an *in vivo* model using *Caenorhabditis elegans* as a host organism for the co-infection, where filamentation of *C. albicans* was inhibited, yet the worm was killed by the infection [138]. The bacteriocin EntV secreted by *E. faecalis* has been recently identified as the compound inhibiting the yeast-to-hyphae transition of *C. albicans*, resulting in decreased virulence and biofilm formation [140,141]. This small peptide was also able to degrade mature fungal biofilms and reduce the virulence of *C. albicans* in a mouse model [141]. In another study, *E. faecalis* was found to produce a non-hemolytic anti-*Candida* protein [142]. Adhesion of *C. albicans* to *E. faecalis* (and other bacteria, such as *Streptococci*), is mediated by the cell wall-associated, secreted aspartyl proteinase Sap9, playing an important role in biofilm development [143]. This suggests that factors produced by *E. faecalis* could be exploited as adjuvants for treatment of *Candida* infection. Additionally, inhibitors of adhesion and biofilm formation may also attenuate virulence and prevent invasive infection by *Candida*.

*Candida albicans* and *Lactobacillus* spp. (Figure 1): The bacterium displays inhibitory effects against *C. albicans.* This interaction is relevant in the female reproductive tract, which is populated by *Lactobacilli* under physiological conditions. The bacteria counteract colonization by *C. albicans* by reducing adhesion of the fungus to epithelial cells. This is achieved via outcompeting the fungal cells for adhesion sites or by decreasing fungal binding through surlactin, a biosurfactant secreted by the bacteria [8,144]. *Lactobacillus* species are able to inhibit hyphal formation of *C. albicans* through soluble metabolic products, short-chain fatty acids (SCFA), H_2_O_2_, and lactic acid. Under glucose-limiting conditions, which occur as a result of carbon deprivation during alkalinization, similar to the conditions existing in phagocytic cells, *C. albicans* can raise the environmental pH by excreting ammonia, thereby inducing hyphal formation [145,146,147].

*Candida albicans* and *Escherichia coli* (Figure 1): Murine models have shown that co-infection with *C. albicans* and *E. coli* resulted in synergistic lethality. In this case, endotoxins produced by *E. coli* during the interaction were identified as the key virulence factors contributing to the high mortality [59,60]. In contrast to the observations in murine models, Hall and colleagues reported that, in the human host, the fungus can apparently suppress the growth of *E. coli*, either directly or indirectly, by dominant interaction during colonization. Under physiological conditions, this effect could possibly inhibit migration of *E. coli* from the rectum to the vaginal area, thereby offering protection against urinary tract infections caused by *E. coli* and other bacteria [58]. However, more studies are required to assess the net effects of this interaction in the clinical setting.

*Candida albicans* and *Actinomyces* spp. (Figure 1): The fungus is able to adhere to different species of *Actinomyces,* which are part of the oral bacterial flora, but *in vitro* studies have shown that the level of aggregation is dependent on the *C. albicans* strain and the culture medium [148,149,150]. This interaction is mediated by a protein on the *Candida* surface that interacts with carbohydrate molecules on the surface of *Actinomyces.* This association results in enhanced cariogenic virulence promoted by increased adhesion, increased biofilm formation, and decreased pH, contributing to oral colonization and oral candidiasis [149,151].

*Candida albicans* and *Acinetobacter baumannii* (Figure 1): Association of these microorganisms has been found in clinical isolates from intensive care units [152], and their interaction displays mutually inhibitory effects. While *A. baumannii* affects hyphal growth of *C. albicans* and can induce apoptotic cell death via contact-dependent signals mediated by the outer-membrane protein A (OmpA) [153,154], *C. albicans* responds to the inhibition of filamentation by suppressing the growth of *A. baumannii* [153]. This effect is conveyed by the secretion of farnesol, which inhibits biofilm formation and reduces the viability of *A. baumannii* [152,155]. The actual clinical impact is difficult to assess, as clinical studies from different intensive care units (ICUs) have indicated a considerable variation in the rate of invasive infections [156].

*Candida albicans* and *Aggregatibacter actinomycetemcomitans* (Figure 1): The bacterium is a Gram-negative opportunistic pathogen causing oral diseases. It produces the quorum-sensing molecule autoinducer-2 (AI-2), inhibiting hyphal structures and biofilm formation of *C. albicans in vitro* [157]. However, the inhibitory interaction observed *in vitro* was not confirmed in the clinical setting. Concomitant isolation of these two microorganisms in women using oral contraceptives has been associated with moderate to severe periodontitis, rather supporting a synergistic effect of the interaction [158]. Despite the questionable association of *Candida* infection with periodontitis, the presence of saliva, which is a strong inducer of hyphal formation, might be responsible for a synergistic effect of the co-infection [157,159]. However, the net effect of this interaction may depend on the amount of bacterial AI-2 produced in saliva-fed biofilms.

*Candida albicans* and *Serratia marcescens* (Figure 1): Although the mechanism of interaction has not been identified to date, *C. albicans* apparently displays a stimulatory effect on the Gram-negative bacterium, enhancing its virulence. This effect has been documented in the peritoneal cavity, and was shown to promote dissemination of the bacterium to several abdominal organs. A similar stimulatory effect was also documented for other bacterial species, including *S. aureus* and *S. faecalis* [100,160]. This is of particular importance in immunocompromised patients, in whom disseminated infection may result in severe sepsis.

*Candida albicans, Candida dubliniensis,* and *Fusobacterium* spp. (Figure 1): The indicated fungi adhere well to several species of *Fusobacterium*, including, for example, *F. nucleatum*, *F. periodontium,* and *F. sulci*, which are colonizers of the oral mucosa [161,162,163]. The aggregation, resulting in mutual inhibition, is thought to be mediated by bacterial lectins, which may interact with carbohydrates on the cell wall surface of *Candida* [161,162]. Recently, additional mediators promoting aggregation of these two microorganisms were identified, involving the bacterial membrane protein RadD, and the *Candida* adhesin-like cell wall mannoprotein Flo9 [164]. Mutual adherence was only observed in the presence of the hyphal form, but not in the yeast form of *C. albicans* [165]. However, the strong co-aggregation could be inhibited by externally added arginine and mannose, which disrupt the proteins RadD and Flo9, respectively [165]. The level of co-aggregation and the inhibitory effects were shown to be strain-dependent [163]. Moreover, the effect of the bacterial–fungal interaction *in vitro* may also be influenced by the growth conditions. Recently, the proteins RadD and Flo9 were found to be involved in the inhibition of hyphal formation of *C. albicans* under specific growth conditions [164]. Growth and filamentation of *C. albicans* were found to be inhibited by *F. nucleatum* in a contact-dependent process [164,165]. These studies suggest that the association between *Candida* and *Fusobacterium* may permit a long-term commensal state in the oral mucosa [164]. However, more studies are needed to assess the impact of this bacterial–fungal interaction in the host, including studies in *in vivo* models.

*Candida albicans* and *Burkholderia* spp. (Figure 1): The bacterium *B. cenocepacia* is an opportunistic pathogen found in the respiratory tract. It is mostly acquired from the environment, via hospital devices or by person-to-person spread, and is only rarely carried as a commensal microorganism [166,167]. *B. cenocepacia* produces a quorum-sensing molecule termed *cis*-2-dodecenoic acid (BDSF), which inhibits initiation of hyphal formation in *C. albicans* [168]. This molecule can also inhibit adherence of *C. albicans* to urinary catheters, as revealed by *in vitro* models [169].

*Candida albicans* and *Clostridium* spp. (Figure 1): These Gram-positive bacteria are obligate anaerobes, and the growth of certain species is promoted by *C. albicans* under hypoxic conditions [170]. However, the presence of *C. albicans* can also be exploited by *Clostridium difficile* to facilitate its growth under aerobic conditions [171]. It has been suggested that *C. albicans* may use its metabolism to reduce the oxygen tension or produce antioxidants such as tyrosol, which would favor the growth of anaerobic bacteria [172,173]. This indicates that *C. albicans* may promote the growth of strictly anaerobic bacteria within oxygen-rich environments [174]. Conversely, *C. difficile* produces *p*-Cresol, a fermentation product derived from tyrosine, displaying inhibitory effects on hyphal formation of *C. albicans*. The compound induces hypha-to-yeast transition, and inhibits biofilm formation and virulence of *C. albicans* [171]. These observations raise the possibility that treatment approaches affecting the aerobic vs. anaerobic environmental conditions may favor the growth of a certain pathogen. Moreover, these studies suggest that patients harboring a *C. difficile* infection might be less prone to developing a systemic *Candida* infection. Conversely, however, elimination of the bacterium by appropriate treatment could promote expansion of the fungus.

*Candida albicans* and *Bacteroides fragilis* (Figure 1): Current knowledge on the interaction between these microorganisms is restricted to the observation that growth of the Gram-negative bacterium *B. fragilis* is promoted by *C. albicans* under aerobic conditions [170].

*Candida albicans* and *Salmonella enterica* (Figure 1): The serovar *typhimurium* of *S. enterica* has been described as inhibiting growth, hyphal formation, and viability of *C. albicans*. It has been suggested that a quorum-sensing molecule secreted by the bacterium might be responsible for this effect [175], and recent data indicate that it is apparently mediated by inositol phosphatase (sopB), an effector of the type III secretion system [176].

*Candida albicans* and *Klebsiella pneumoniae* (Figure 1): Antagonistic interactions between the Gram-negative bacterium *K. pneumoniae* and *C. albicans* have been reported. The bacterium adheres to both yeast and hyphal structures of *C. albicans*, and inhibits growth of the fungus. However, the specific mechanisms of this interaction have not yet been elucidated [170].

#### 3.1.2. *Aspergillus* Species and Bacteria 

**Figure 2:**
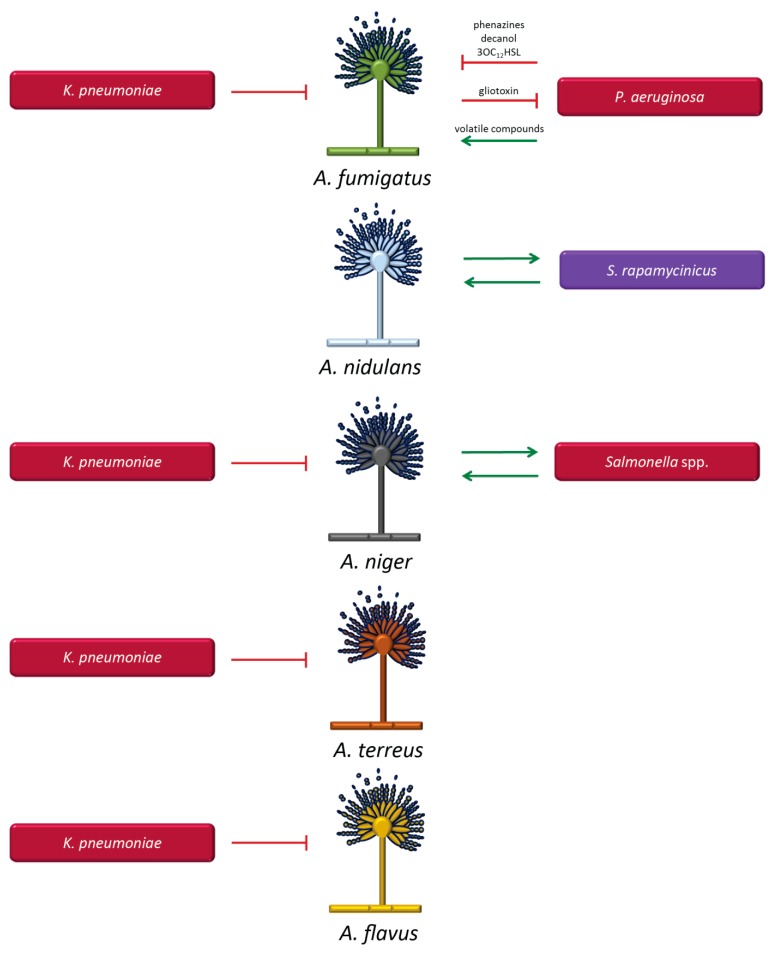
Molecules and factors mediating the interaction between *Aspergillus* species and bacteria. *Aspergillus* species include *Aspergillus (A.) fumigatus*, *Aspergillus (A.) nidulans*, *Aspergillus (A.) niger*, *Aspergillus (A.) terreus* and *Aspergillus (A.) flavus*. Gram-positive bacteria are represented in lilac (*Streptomyces (S.) rapamycinicus*) and Gram-negative bacteria in red (*Klebsiella (K.) pneumoniae*, *Pseudomonas (P.) aeruginosa*). Green arrows indicate supportive interactions and red lines represent inhibitory effects. If not indicated above the green arrows and red lines, the molecules mediating the interaction are currently unknown.

*Aspergillus fumigatus* and *Pseudomonas aeruginosa* (Figure 2): Co-localization of *A. fumigatus* and *P. aeruginosa* in the lungs of patients with cystic fibrosis has been associated with poorer outcomes when compared to single infections with these pathogens [53,85]. *P. aeruginosa* has the capacity to inhibit the growth of *A. fumigatus* [177,178,179,180]. This interaction occurs through the production of quorum-sensing molecules and virulence factors by *P. aeruginosa,* including, for example, phenazines, decanol, and 3-oxo-C12-homoserine lactone (3OC_12_HSL), which affect hyphal development through the generation of highly toxic reactive oxygen species (ROS) [179,181,182]. Moreover, the inhibitory effect also involves the phenazine derivatives, pyrrolnitrin and pyocyanin [182], and the LasIR quorum sensing system has been implicated in inhibiting *A. fumigatus* biofilms [181]. Conversely, *A. fumigatus* was recently found to be able to inhibit *P. aeruginosa* in mixed culture, leading to reduced biofilm formation. The compound gliotoxin produced by *A. fumigatus* was identified as the main agent responsible for the inhibitory effect. In addition, iron regulation also plays a key role in this interaction. *A. fumigatus* produces siderophores that help the fungus protect itself against iron-chelation by *P. aeruginosa* [183]. The indicated interactions are mutually antagonistic [180], thus failing to provide a rational explanation for the clinical impact of the co-infection, but *P. aeruginosa* also has the capability to produce volatile compounds that stimulate the growth of *A. fumigatus* at a distance rather than by direct contact [184].

*Aspergillus nidulans* and *Streptomyces rapamycinicus* (Figure 2): The Gram-positive bacteria of the genus *Streptomyces* are normally encountered in soil. Although infections in humans are rare, *Streptomyces* can activate genes of secondary fungal metabolism, including those responsible for synthesis of antibiotic and aromatic polyketides [185]. The activation requires physical contact between *A. nidulans* and *S. rapamycinicus* [186]. This interaction is thought to reflect a symbiotic relationship, with activation of silent gene clusters in the fungus mediated by chromatin remodelling [185]. Recent findings suggest a new transcription factor, BasR, as a key regulator for the transduction of the bacterial signal [187]. The findings suggest that infections with *Streptomyces* may change the local environment and alter the microbial composition by activating fungal-derived synthesis of antibiotic compounds.

*Aspergillus niger* and *Salmonella* spp. (Figure 2): The interaction between *A. niger* and *Salmonella* spp., including all serovars of *S. enterica*, is mediated by attachment of the bacterial cellulose to the fungal cell wall component chitin on hyphae of *A. niger*, promoting the formation of multi-layered and branched biofilms [188]. Although the potential clinical consequences of this interaction have not been elucidated, it is conceivable that the enhanced capacity to bind fungi via cellulose production may result in stronger biofilm formation, thus contributing to increased antimicrobial resistance. Cellulose production by pathogenic bacteria may therefore constitute a survival advantage.

*Aspergillus* spp. and *Klebsiella pneumoniae* (Figure 2): Recently, studies performed in our laboratory have shown that *K. pneumoniae* exerts an inhibitory effect on *Aspergillus* species, including *A. fumigatus, A. terreus, A. niger,* and *A. flavus*. *Aspergillus* spore germination was inhibited, as well as the development of pre-formed hyphal structures. *K. pneumoniae* also significantly decreased biofilm formation of *Aspergillus* species [189]. The exact mechanisms and molecules involved in this interaction are currently under investigation. The clinical impact of this bacterial–fungal interaction may be of particular importance in the setting of cystic fibrosis and other lung-associated diseases because both pathogens co-habit in the lungs.

#### 3.1.3. Cryptococcus Species and Bacteria 

**Figure 3:**
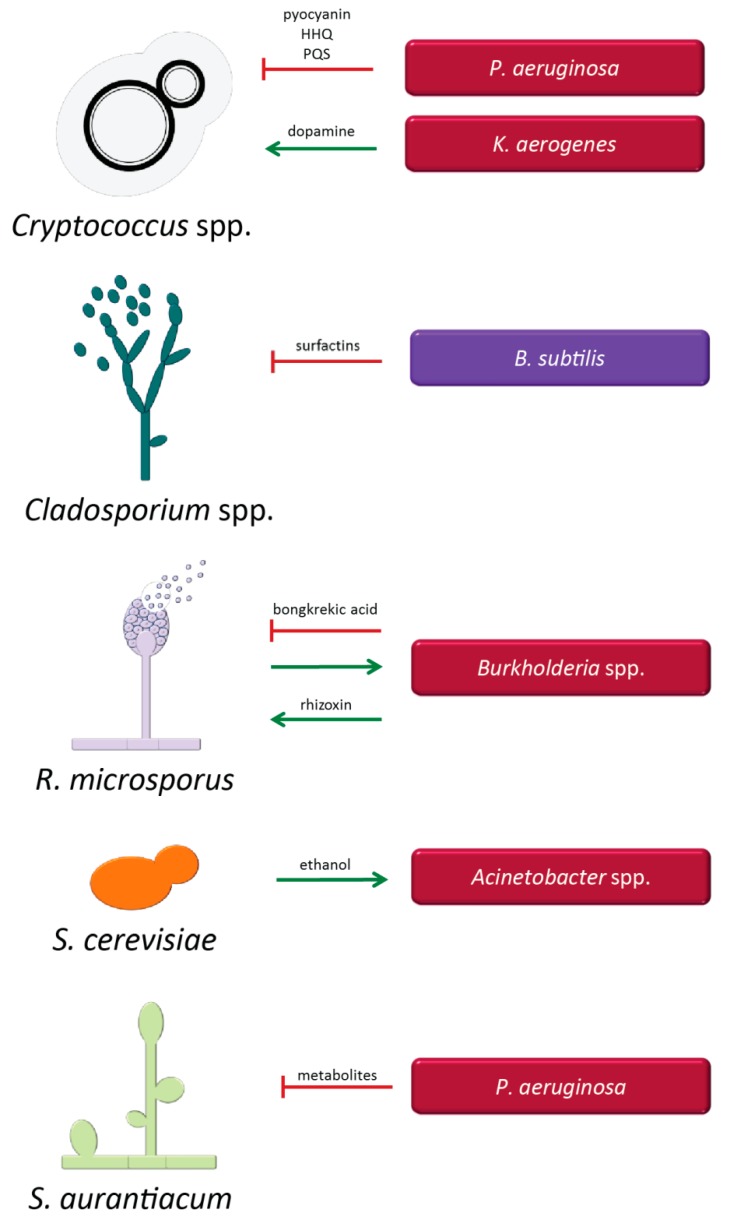
Molecules and factors mediating the interaction between *Cryptococcus* spp., *Cladosporium* spp., *Rhizopus microsporus, Saccharomyces cerevisiae, Scedosporium aurantiacum*, and different bacteria. Gram- positive bacteria are represented in lilac (*Bacillus (B.) subtilis*) and Gram-negative bacteria are represented in red (*Pseudomonas (P.) aeruginosa, Klebsiella (K.) aerogenes*). Green arrows indicate supportive interactions and red lines represent inhibitory effects. If not indicated above the green arrows and red lines, the molecules mediating the interaction are currently unknown.

*Cryptococcus* spp. and *Pseudomonas aeruginosa* (Figure 3): The lungs of immunocompromised patients commonly display the concomitant presence of *Cryptococcus* spp., including in particular *C. neoformans*, and *P. aeruginosa,* and the bacterium has the capacity to inhibit the growth of *Cryptococcus* spp. [190,191]. The inhibitory effect occurs mainly through the production of the metabolite pyocyanin, but also by the production of alkylquinolones such as HHQ (4-hydroxy-2-heptylquinoline) and PQS (3,4-dihydroxy-2-heptylquinoline) [105,106,190,191]. Cell contact is necessary for maximum inhibition of *Cryptococcus* growth [190]. The clinical impact of this interaction is currently unknown.

*Cryptococcus neoformans* and *Klebsiella aerogenes* (Figure 3): This bacterial species induces melanin production by *C. neoformans* through secretion of dopamine by the bacterium, thereby leading to enhanced protection of *C. neoformans* from macrophages [192,193]. Enhanced melanization of *Cryptococcus* may also confer a protective effect against antifungal treatment.

#### 3.1.4. Interaction of Other Fungal Species with Bacteria 

*Cladosporium* spp. and *Bacillus subtilis* (Figure 3): Different species of the mould *Cladosporium* are involved in mediating allergic reactions, particularly in individuals with pre-existent respiratory diseases, and can also cause infections of the skin, sinuses, and lungs [194]. A class of diphenyl ethers with polyhydroxy sidechains has been identified when *Cladosporium* species and *B. subtilis* interacted *in vitro*, and it was suggested that the production of these compounds may be a defensive response of the fungus against growth inhibition mediated by *B. subtilis* through the secretion of surfactins (antifungal cyclopeptides). Surfactins have been suggested to cause the induction of secondary metabolism in *Cladosporium* spp. [195]. Activation of secondary metabolism and production of specific metabolites may result in increased fitness and virulence of these fungal species in the host.

*Rhizopus microsporus* and *Burkholderia* spp. (Figure 3): Fungi belonging to the genus *Rhizopus* cause infections known as zygomycosis. The fungus *R. microsporus* and the bacterium *Burkholderia gladioli* are plant pathogens but can also cause opportunistic infections in humans [196]. Upon interaction with *R. microsporus*, *B. gladioli* produces the compound bongkrekic acid, which acts as a respiratory toxin, but also results in fungal growth inhibition [197,198]. *R. microsporus* indirectly contributes to production of bongkrekic acid by stimulating bacterial growth [197]. *R. microsporus* can also establish a symbiotic interaction with *B. rhizoxinica.* This endosymbiotic bacterium produces an important compound, rhizoxin, which is essential for fungal spore formation, and is also considered as a mediator of antitumor activity [199]. It is crucial, therefore to assess which pathogens can cause infections in humans and, in the presence of polymicrobial interactions, to identify factors capable of affecting concomitant treatment approaches.

*Saccharomyces cerevisiae* and *Acinetobacter* spp. (Figure 3): The fungus produces ethanol, which can promote growth of several *Acinetobacter* species, including *A. baumannii, A. haemolyticus, A. johnsonii*, and *A. radioresistens in vitro* [200]. As demonstrated for *A. baumannii* in a *C. elegans in vivo* model, these BFI can result in increased bacterial resistance to osmotic stress associated with enhanced pathogenicity and virulence [200]. Improved fitness of these bacteria might be of particular interest in patients with high alcohol consumption [201], and could ultimately affect the responsiveness to antimicrobial treatment. 

*Scedosporium aurantiacum* and *Pseudomonas aeruginosa* (Figure 3): The filamentous fungus *S. aurantiacum* is an opportunistic pathogen that can be isolated from the lungs of patients with cystic fibrosis. Interactions between this fungus and *P. aeruginosa,* one of the most important bacteria in this disease, were shown to be inhibitory for the fungus. The effect does not require biofilm formation involving *P. aeruginosa,* but metabolites secreted by the bacterium are suspected to be responsible for the inhibitory interaction. Pyocyanin, a molecule commonly secreted by *P. aeruginosa,* showed no effect against the fungus [202,203] and the mediators of inhibition, and thus the potential clinical impact of the interaction remains obscure.

## 4. Host Immune Response to Bacterial and Fungal (Co-)Infections

Perturbations of the microbiome and weakening of the host immune system are conditions facilitating the transition of opportunistic microbes from a commensal to a pathogenic state, mediating the initiation of infection. Microbiota can also influence gene expression of mucins and toll-like receptors (TLRs) by the host, and mediate modulation of the immune system and apoptosis [32]. Factors predisposing the human host for invasive fungal infections include i) long-term or repeated exposure to broad-spectrum antibiotics; ii) impairment of epithelial barriers affecting the skin, the gastrointestinal tract, or other mucous membranes, e.g., by chemotherapy, surgery or central venous catheters; and iii) treatment with immunosuppressive agents such as corticosteroids [204].

The interaction between microbial pathogens and the host induces the activation of several virulence factors and adaptation mechanisms [204]. In fungal infections, the virulence factors include morphological transitions (e.g. yeast-to-hyphae), phenotypic switching (e.g., white to opaque state in *C. albicans*), biofilm formation, increased adhesion capacity, and environmental pH modulation [205].

The first line of defense against fungal pathogens is mediated by the innate immune response. Pattern recognition receptors (PRRs) expressed on the surface of immune cells recognize pathogen-associated molecular patterns (PAMPs) which comprise several cell wall components, such as mannans, mannoproteins, β-glucans, and chitin, as well as fungal-derived RNA and unmethylated DNA [206]. PRRs include toll-like receptors (TLRs) and C-type lectin receptors (CLRs), which are present on macrophages and dendritic cells. Upon ligand binding, the immune response is initiated through a series of signaling cascades, which, in turn, result in fungal internalization via phagocytosis, and production of cytokines and reactive nitrogen and oxygen species (RNS and ROS) [206]. Most of the fungal cell wall components can be recognized by TLR2, TLR4, and TLR9, which trigger the activation of dendritic cells and transcription of proinflammatory cytokines (IL-1β, IL-6, IL-23) [207,208]. Proinflammatory cytokines bind to receptors on Th17 cells [208]. Neutrophils play a key role against bacterial pathogens by producing large amounts of cytotoxic ROS, proteases, and antimicrobial peptides [209,210], but also play an essential role in the defense against fungal infections. In response to chemotactic factors released by the pathogens and the host, neutrophils rapidly migrate to the infection site, and neutropenia is therefore a risk factor for both fungal and bacterial infections associated with adverse clinical outcome [204,211]. However, excessive accumulation of neutrophils in the course of an infection leads to increased tissue damage, underlining the potential pathogenic effect of the immune response [204]. Synergistic associations between different PRRs (e.g., TLR2 and Dectin-1) have also been found to facilitate the PAMP recognition and to enhance downstream responses [204]. However, the immune response is not always effective. Fungi have developed several mechanisms and strategies to escape the attack of the immune system. The escape mechanisms essentially include shielding of PAMPs through the cell wall or capsule, and the formation of biofilms, titan cells, asteroid bodies, or dimorphism (yeast-to-hyphae transition). For example, virulence factors of *C. albicans* are exclusively expressed at the hyphal stage, and hyphal cells induce low cytokine production compared to yeast cells. Hyphal structures are also important to evade phagocytosis and escape from the immune cells [211].

Bacteria have also developed mechanisms to hide or escape from the immune system. Some of these mechanisms are similar to those used by fungi. Biofilm formation is also an important feature used by bacteria to evade the immune response. Other factors include the secretion of proteins, quorum sensing regulation, production of antigenic exotoxins, pore-forming toxins, and capsular polysaccharides. Different capsular serotypes exist in bacteria and differ in their chemistry and antigenicity [210,212,213]. Capsular polysaccharides minimize or even inhibit the host recognition, either by hiding or modifying the cell surface. Bacteria can also dampen opsonization through the expression of proteins on the cell surface or by their secretion. Recruitment of neutrophils to the infection site can be inhibited, and killing of neutrophils may occur through the secretion of toxins or cytolysins [210]. Moreover, bacterial secretion systems may also be used to inject effector proteins directly into the host cells, including immune cells [214], and bacteria have developed mechanisms to manipulate the inflammatory pathways, induce immune cell death (apoptosis, pyroptosis), and tolerate different pH conditions [212,215]. Escape from the immune system may lead to persistent and chronic infection, bearing the risk of potentially life-threatening reactivation occurring particularly in severely immunocompromised individuals.

## 5. *In Vivo* Models of Bacterial and Fungal (Co)-Infections

Bacterial–fungal interactions can display a diverse spectrum of effects, which may not be identical *in vivo* and *in vitro*. For example, the interactions between *C. albicans* and *P. aeruginosa* observed in *in vitro* models are mostly antagonistic, but the interaction in the human host displays synergistic effects on the virulence, resulting in higher mortality [1,2,216]. The differential effect may be explained by the host environment and the increased inflammatory response associated with cytokine profiles that are absent *in vitro* and also differ from single pathogen infections (Table 1). Co-infection with *C. albicans* and *P. aeruginosa* revealed significant upregulation of the proinflammatory cytokine IL-6 and a less prominent increase of IL-8, a potent chemoattractant of neutrophils in a zebrafish *in vivo* model [2]. However, other studies in mice have shown that infection with *C. albicans* mediates a protective effect against lung tissue damage induced by *P. aeruginosa*. This effect reportedly occurs by triggering IL-22 production, activation of the IL-17 pathway, and via stimulating the production of antimicrobial peptides by the host [99]. Similarly, co-infection of *P. aeruginosa* and *A. fumigatus* has also been described as resulting in poorer outcomes in cystic fibrosis patients, in comparison to infections by single-pathogens [53]. In contrast to the observations in the human host, no additive effect on the inflammatory response was observed in corresponding co-culture experiments in the wax moth *Galleria mellonella* model. The lack of synergistic inflammatory response in epithelial cells of cystic fibrosis patients may be explained by the saturation of signaling pathways for cytokine production, since both organisms activate the same pathways [180]. *Streptococcus* species are very important colonizers of the oral mucosa. A co-infection with *C. albicans* is synergistically pathogenic in a murine model, leading to the formation of hypervirulent mucosal biofilms [113,217], and the inflammatory response has been shown to be dependent on TLR-2 signalling, with specific cytokine and genetic signatures associated with this co-infection [113].

Single pathogen infection with *S. aureus* was shown to be avirulent in a mouse model, whereas co-infection with *C. albicans* resulted in 100% mortality within 48–72 h post inoculation [218]. Similar observations were also made in a corresponding co-infection model using *G. mellonella* or *C. elegans*, where enhanced pathogenicity and increased mortality was observed [219,220]. However, the mortality rate in the mouse model was apparently dependent on the *Candida* species involved, as co-infections of *S. aureus* with *C. dubliniensis*, *Candida parapsilosis*, or *C. glabrata* resulted in low or no mortality at all [218]. During these co-infections involving *C. albicans* and *Candida krusei*, IL-6 and prostaglandin E2 (PGE2) were found to be significantly elevated, which was not the case in co-infections with other *Candida* species [218]. 

Co-infection of *C. albicans* and *E. coli* also resulted in 100% mortality in a murine model, compared to only 3% and 20% mortality of single infections by *C. albicans* and *E. coli*, respectively [59], and the bacterial endotoxins produced during the co-infection are thought to mediate the synergistic effect on mortality [59].

Surprisingly, in a *C. elegans* model of co-infection with *C. albicans* and *E. faecalis*, the worms lived much longer than upon infection with *C. albicans* only, and this effect was even more dramatic upon sequential exposure to *E. faecalis* followed by *C. albicans*. It is conceivable that priming the host immune system with *E. faecalis* somehow protected the worm against subsequent exposure to *C. albicans*, and the effect is thought to be due to the inhibition of *C. albicans* filamentation, thereby greatly reducing tissue damage in the worm [138,140]. A similar effect was observed in co-infection with *C. albicans* and *A. baumannii* in a *C. elegans* model, where inhibition of *C. albicans* filamentation by *A. baumannii* attenuated the pathogenicity of the fungus, leading to reduced lethality [153].

For many of the bacterial–fungal interactions described above, no data are available regarding the interplay with the host and how the immune system responds to such polymicrobial infections compared to the respective single infections. In Table 1, important features of the bacterial–fungal interactions and the interplay with the host immune system are summarized. It is important to emphasize again that bacterial–fungal interactions observed *in vitro* can greatly differ from the observations made *in vivo,* either in model animal systems or in the human host. The immune response, including the degree of inflammation, can exert a major effect on factors affecting the pathogenicity and virulence of the individual pathogens involved, and may thus also affect the overall result of the bacterial–fungal interaction.

## 6. Conclusions

Current data underline the importance of identifying polymicrobial infections involving bacteria and fungi, and taking their possible interactions into consideration as a basis for efficient diagnostics and treatment. Detailed knowledge of clinically relevant bacterial–fungal interactions not yet characterized to date is needed, with particular emphasis on deciphering the ways of communication between multidrug resistant pathogens such as *Candida auris* or methicillin-resistant *Staphylococcus aureus* (MRSA). Employment of state-of-the-art technologies, including CRISPR-Cas (clustered regularly interspaced short palindromic repeats) gene editing and mutant libraries, will facilitate the identification of key regulators mediating bacterial–fungal interactions and their interplay with the host immune system. It is necessary to bear in mind, however, that the effects of BFI observed in assays performed *in vitro* can be variable, depending on the experimental conditions, and the results can be discordant with those obtained in different *in vivo* models, thus rendering interpretation of the clinical relevance challenging.

Production of certain metabolites during BFI may result in increased fitness and virulence of the microorganisms involved. As described in this review, bacteria such as *Streptococcus* spp. can promote hyphal development in fungal species. Hyphal structures play a crucial role in the invasion of epithelial cells and organs, thus promoting expansion of the infection. Moreover, hyphae provide better fitness under challenging environmental conditions, mediate increased adhesion properties, and permit strong biofilm development, which is associated with increased antimicrobial resistance. Importantly, dual-species biofilms have shown increased resistance to drug treatment compared to single-species biofilms [219]. Hyphal development, cell adhesion, and biofilm formation often serve as targets for treatment of monoinfections. Since these factors are also affected during many of the bacterial–fungal interactions studied, they may also serve as targets for appropriate treatment strategies in polymicrobial infections. Some authors have argued that specific classes of antifungal drugs, including echinocandins in particular, might prove beneficial for treating or preventing polymicrobial infections by exerting immunomodulatory properties. However, this immune potentiation is apparently non-specific as it also occurs in response to monoinfections [230].

New diagnostic approaches based on the identification and exploitation of novel biomarkers for BFI that will expectedly emanate from ongoing research are required for appropriate management of polymicrobial infections. Diagnostic biomarkers for pathogenetically relevant processes occurring during BFI, including adhesion, development of hyphal structures, and mixed biofilm formation, are crucial for the development of new adjuvant therapy approaches complementing the use of established treatment strategies with antibiotic and antifungal drugs. Therapeutic interference with the communication between bacterial and fungal pathogens, as well as control of exacerbated inflammatory response, could be potential targets for improved control of specific polymicrobial infections. Novel therapeutic approaches targeting quorum sensing and microbial metabolites may be devised to tackle both bacterial and fungal infections [87,88,89,90,91,92,93,94,95] in combination with antibiotic and antifungal drugs to enhance the efficacy of treatment [96,97,98]. New insights acquired in this field will expectedly pave the way for more efficient personalized treatment strategies.

## Figures and Tables

**Table 1:** Animal models used for studies of bacterial–fungal interactions and immune response.

Bacterial–Fungal Interaction	Host		Immune Response		References
	*In Vivo* Model	Mortality	Cytokines/Chemokines/Molecules	Effect	
*Candida albicans* and *Pseudomonas aeruginosa*	Rat		Elevated pro-inflammatory cytokines: TNF-α, IFN-γ, IL-6	Higher bacterial loads in the lungs; impaired macrophage function in the lungs	[216]
	Mouse	↑		High mortality mostly due to protease activity of *P. aeruginosa*	[1]
	Mouse	↓	IL-22; IL-17 pathway; AMPs	Colonization by *C. albicans* leads to protection against *P. aeruginosa-*associated pneumonia; lower bacterial loads and decreased epithelial injury	[99,221]
	Mouse	↓		Factors secreted by *C. albicans* inhibit *P. aeruginosa* siderophores via cytotoxic molecules reducing the bacterial virulence	[222]
	Zebrafish	↑	Elevated pro-inflammatory cytokine: IL-6; Elevated neutrophil chemoattractant: IL-8	Increased *C. albicans*–mediated pathogenicity and virulence; increased inflammatory response; no excessive neutrophil infiltration	[2]
*C. albicans* and *Streptococcus* spp.	Mouse		Toll-like receptor (TLR)-2 signalling; cytokines IL-17C, CXCL1, MIP-2/CXCL2, TNF, IL-1α, IL-1β; neutrophil protein CD177, CD14, MMP8	Increased *C. albicans*–mediated pathogenicity and virulence; increased inflammatory response; increased neutrophil infiltration; hypervirulent biofilms; hyphal gene *EFG1* required for robust mixed biofilms	[113,223]
*Candida* spp. and *Staphylococcus* spp.	Mouse	↑	IL-6; PGE2; IL-1β; TNF-α	Yeast-to-hyphae transition of *Candida* does not influence dissemination and lethal sepsis	[218,224]
	*C. elegans*	↑	C-type lectins; CUB domain containing factors; AMPs	Increased virulence of both species	[220,225]
	*Galleria mellonella*	↑		Increased pathogenicity; *Staphylococcus aureus* helps *C. albicans* circumvent the IS, contributing to its persistence	[219,226]
*C. albicans* and *Enterococcus faecalis*	*C. elegans*	↓		Exposure to *E. faecalis* primes the IS to better cope with later exposure to *C. albicans*; hyphae are inhibited; reduced tissue damage	[138,140]
*C. albicans* and *Escherichia coli*	Mouse	↑	Endotoxin mediating synergistic lethality	Currently unknown	[59,60]
*C. albicans* and *Lactobacillus* spp.	Mouse	↓	TNF-α; IFN-γ; IL-6; IL-10; IL-22	Bacterial treatment followed by *C. albicans* infection improved survival and resistance of the mouse	[227,228]
*C. albicans* and *Acinetobacter baumannii*	*C. elegans*	↓		*C. albicans* pathogenicity is decreased; hyphae are inhibited; *C. albicans* proliferation in the gut is reduced	[153]
*Aspergillus fumigatus* and *P. aeruginosa*	*G. mellonella*	↑	Activation of mitogen-activated protein kinases (MAPKs) ERK and p38	No additive of the co-infection on inflammation; lack of synergistic inflammatory response; saturation of signaling pathways	[180]

The documented mortality of these bacterial–fungal interactions (BFI) is presented as higher (upward arrows) or lower (downward arrows) compared to the respective single infections. Cytokines, chemokines, and other molecules involved during the BFI, the immune system interaction and a brief description of the mechanisms are indicated. PGE2—prostaglandin E2; IS—immune system; CUB—C1s/C1r complement components, the embryonic sea urchin protein (Uegf), and bone morphogenetic protein 1 (Bmp1) [229]; AMPs—antimicrobial peptides.
